# Vaccine Type-, Age- and Past Infection-Dependence of the Humoral Response to SARS-CoV-2 Spike S Protein

**DOI:** 10.3389/fimmu.2022.809285

**Published:** 2022-02-28

**Authors:** Salvador Romero-Pinedo, Marina Quesada, Lydia Horndler, Stela Álvarez-Fernández, Asunción Olmo, David Abia, Balbino Alarcón, Pilar Delgado

**Affiliations:** ^1^ VITRO S.A., Granada, Spain; ^2^ Centro de Biología Molecular Severo Ochoa, Consejo Superior de Investigaciones Científicas (CSIC), Universidad Autónoma de Madrid, Madrid, Spain

**Keywords:** SARS-CoV-2, COVID-19, antibody response, humoral immunity, vaccines

## Abstract

The emergence of COVID-19 has led to a worldwide challenge for the rapid development of vaccines. Several types of safe and effective vaccines have been available in a time frame never seen before. Now that several hundred million people have been vaccinated there is an opportunity to compare vaccines in terms of protection and immune response. Here, we have applied a highly sensitive multiplexed flow cytometry method to measure simultaneously IgM, IgG1 and IgA anti-spike protein antibodies generated in response to three vaccines: ChAdOx1 (Oxford-AstraZeneca), mRNA-1273 (Moderna), and BNT162b2 (Pfizer-BioNTech). We have found that mRNA vaccines (mRNA-1273 and BNT162b2) induce a stronger humoral response, both after the first and the second dose, than the adenovirus-based ChAdOx1 vaccine. We also found that, in the elderly, antibody titers negatively correlate with the age of the donor but, also, that antibody titers remain stable for at least 6 months after complete vaccination. Finally, we found that one dose of BNT162b2 is sufficient to induce the highest antibody titers in seropositive pre-vaccination donors. We hope these data will help to guide future decisions on vaccination strategies.

## Highlights

- mRNA-1273 and BNT162b2 RNA elicit higher titers of IgG1 and IgA antibodies for the S protein in serum than adenoviral vaccine ChAdOx1 both after the priming and the booster doses.
- One dose of BNT162b2 to COVID-19-recovered patients is sufficient to produce high titers of anti-S antibodies, higher than those of naïve individual receiving a full vaccination schedule.
- There is an inverse effect of age on the anti-S antibody response to full vaccination with BNT162b2.

## Introduction

SARS-CoV-2 is the virus responsible for the current COVID-19 pandemic. Several vaccines have been quickly and effectively developed to resolve this pandemic (1–3). More than 400 million doses have been administered only in the United States as of early November 2021 (OurWorldInData.org) and studies to investigate the comparative degree of protection provided by the different vaccines still have to be carried out. It is currently accepted that protection correlates with the humoral response (4, 5). Moreover it is much easier to measure antibody than cellular responses and both often correlate to some extent (6). The Spike protein (S), abundant in SARS-CoV-2 viral surface, is highly immunogenic and antibodies against S are already detected one week after infection and lasting one year or more (7, 8). The individual humoral response to the S protein is highly variable (9) and this makes evaluation of the humoral response highly dependent on the reliability and sensitivity of the detection method. ELISA or CLIA are the most frequently used methods for specific antibody quantification. Both rely on detection of few epitopes present in recombinant protein fragments generated in conditions that do not fully reproduce the native status of infected cells (10). We have recently developed a highly sensitive method to detect specific IgG1, IgA and IgM against native SARS-CoV-2 S protein based on flow cytometry (11), from now on named SARS-CoV-2 S Jurkat Flow-Cytometry Immunoassay (JFCI). We have previously demonstrated that our JFCI is superior to ELISA-based methods to detect sera of donors containing neutralizing antibodies. The S protein is expressed on the viral envelope, as well as on the surface of the cells used in the JFCI method, as a trimer. Due to its high sensitivity, JFCI allows to detect specific anti-S antibodies present in blood samples that were undetected by other methods like ELISA or CLIA, which miss the quaternary structure of the S protein (11, 12). In addition to being based on the expression of the S protein in its native form, the JFCI method has another very important advantage over CLIA and ELISA methods, that is the co-expression from the same mRNA and the same polypeptide precursor of both the S protein and a marker protein (either a truncated form of the EGFR or GFP) that allows to implement a clear cut-off value for positive/negative discrimination based on the slope of the S fluorescence intensity cell-per-cell and the fluorescence intensity of the marker. Thus, within the Jurkat cell population, the cells which are brighter for S protein are also brighter for the marker protein, so that plotting the fluorescence intensity for S protein versus the fluorescence intensity for the marker protein of the entire cell population results in a typical diagonal distribution. Negative sera that to not have antibodies against the S protein do not produce those diagonal distributions since antibodies binding non-specifically to Jurkat cells will bind independently of the content in S (and marker) proteins. The JFCI detected as seropositive serum samples that actually contain neutralizing antibodies as it was validated by neutralization of viral particles pseudotyped with the SARS-CoV-2 Spike protein (11). Furthermore the JFCI is multiplexed to detect several Ig isotypes in a single assay.

In this study we have applied the JFCI method to carry out a comparative analysis of humoral SARS-CoV-2 S-specific immune response in volunteers that have been vaccinated with ChAdOx1 (Oxford-AstraZeneca), mRNA-1273 (Moderna) or BNT162b2 (Pfizer-BioNTech). We have found that although the 3 vaccines elicited a detectable humoral response in all blood donors after complete vaccination, there are quantitative differences both in serum IgG1 and IgA. In addition, we found that the magnitude of the antibody response declines with the age of the donor although it lasts up to at least 6 months post-vaccination. Finally, we have found that one dose of BNT162b2 is sufficient to achieve the maximum humoral response in seropositive pre-vaccination donors.

## Materials and Methods

### Cells

The human T-cell line Jurkat clone E6-1 was acquired from ATCC (TIB-152). Jurkat-S-GFP were stablished by transduction with the lentiviral vector based on the epHIV-7 plasmid where the human EGFR reporter was substituted by GFP and the full-length Spike S protein of Wuhan-Hu-1 was cloned (13). Cells were maintained in complete RPMI 1640 (GIBCO) supplemented with 10% fetal bovine serum (Sigma) and 100 U/mL penicillin-Streptomycin (GIBCO) in a humidified air-5% CO_2_ incubator at 37°C. Cells were routinely tested for the absence of mycoplasma.

### Blood Samples and Sera Collection

Individual fingertip blood samples were taken in Microvette^®^200 Capillary Blood Collection tubes (Sarstedt). Then, sera collection was carried out after centrifugation at 10.000 rcf for 5 minutes, maintained at 4°C until use or -20°C for long time conservation. All participants provided written consent to participate in the study which was performed according to the EU guidelines and following the ethical principles of the Declaration of Helsinki. Serum sample collection was included in the study “ACE2 as a biomarker with utility for identification of high risk population for SARSCoV-2 infection and prognosis of evolution in COVID-19” approved by Autonomous University of Madrid Research Ethics Committee, no.2352. A description of samples within each cohort and full data is provided in **Supplementary Table**.

### SARS-CoV-2 S Jurkat Flow-Cytometry Immunoassay (JFCI) Procedure

Jurkat-S-GFP cells were adjusted to 1,2x10^5^ cells per well and plated in 96 well plates. Cells were centrifugated at 282 rcf for 2 minutes at 4°C and the supernatant was eliminated. The cell pellet was resuspended in 150 µl of working buffer that consist in phosphate-buffered saline (PBS), 2% bovine serum albumin (BSA, Sigma Aldrich) and 0,02% sodium azide (Sigma Aldrich) and washed by centrifugation. Then, cells were incubated with sera samples at a dilution of 1:50 with working buffer in a final volume of 100 µl for 20 minutes at 4°C. After incubation cells were washed to eliminate the unbound antibodies and possible interfering materials on the serum. Then cells were stained with 50 µl of the following cocktail of antibodies and viability reagent prepared in working buffer: 1:100 of mouse anti-human IgG1-PE (Ref.: 9054-09, Southern Biotech), 1:100 of mouse anti-human IgM-Pacific Blue (Ref.: PB-320-C100, Exbio), 1:50 of goat anti-human IgA-Alexa Fluor 647 (2052-31, Southern Biotech) and 1:50 of 7-AAD Viability Staining Solution (EXBOO26, Exbio). After incubation two additional washes were carried out. Cells were finally resuspended in the working buffer and analyzed on an Omnicyt™ Acoustic Focusing flow cytometer (Cytognos, S.L.) using the CytKick™ Autosampler (ThermoFisher Scientific). Data were processed with FlowJo software (BD). A pool of sera from 4 seronegative donors (unvaccinated and non-infected) and other pool of sera from 5 seropositive and/or vaccinated donors were used as negative and positive controls respectively (**Supplementary Table**). For each Ig isotype score cut-off values were determined with a collection of pre-COVID-19 sera and serial dilutions of seropositive samples and calculated as previously described (11). Ig to GFP ratio was normalized to 10 for each isotype according to the positive control pool sera.

### Statistics

All data was analyzed using the GraphPad Prism 8 software. Outliers were removed from all data series with the Identify Outliers tool (Rout Q=0.1%). Error bars in figures represent SEM. One- or two-way ANOVA was applied to continuous data following a normal distribution and two-tailed Fisher’s exact test was used to assess differences between categorical variables. Sidak’s correction, with individual variances computed for each comparison, was applied to multiple comparisons performed with ordinary two-way ANOVA. For multiple comparisons with one-way ANOVA, Brown-Forsythe and Barlett’s test was first performed to determine homoscedasticity. Then, Sidak’s correction and ordinary one-way ANOVA was applied to data with equal SD whereas Games-Howell’s correction and Brown-Forsythe ANOVA when the equal SD requisite was not fulfilled. Occasionally, t-test was used for few number of comparisons. Differences were considered statistically significant at P<0.05 (*p <0.05; **p ≤ 0.01; ***p ≤ 0.001; ****p < 0.0001). Serum samples were received coded from the providers and the experimentalists were blinded to their nature until all data analysis was finalized.

## Results

**Figure 1:**
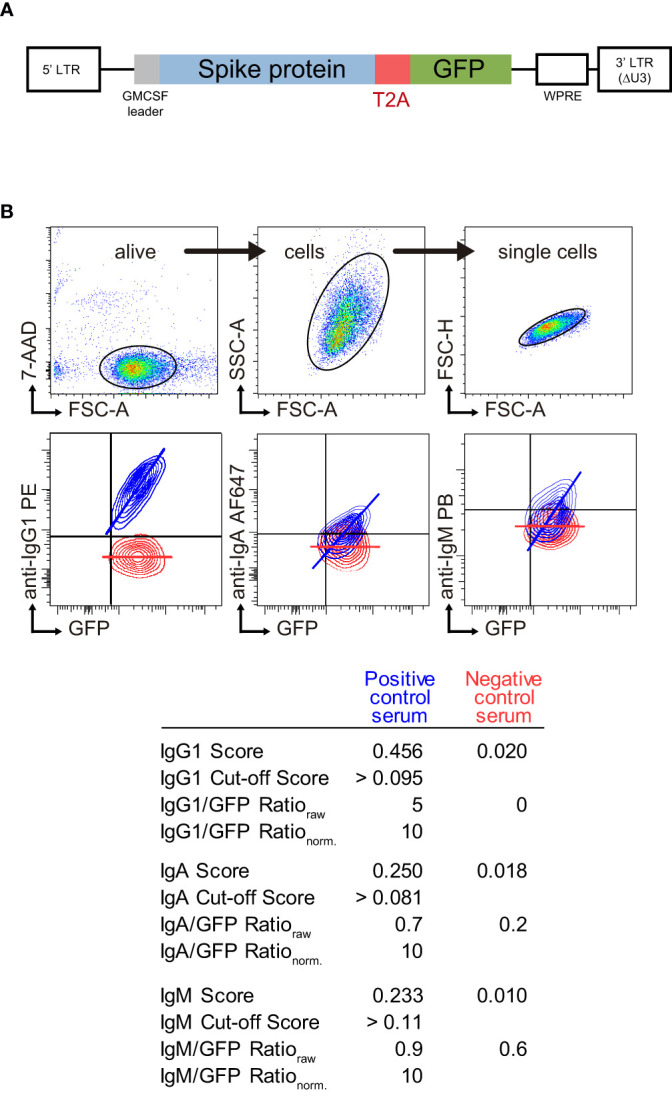
SARS-CoV-2 S Jurkat Flow-Cytometry Immunoassay (JFCI) method. **(A)** Lentiviral construct used to generate Jurkat S GFP cells. Full length mature SARS-CoV-2 Spike protein is followed by the T2A processing viral sequence and GFP. **(B)** Dot plots showing the gating strategy (top) for alive (7-AAD^-^), single cells and overlay contour plots of Jurkat S GFP cells stained with positive (blue) and negative (red) pool of sera (bottom). GFP and the three isotype signals are depicted. Score, cut-off value, raw and normalized ratio for each sample and isotype are indicated in the table below.

We have developed a variant of the JFCI method to detect antibodies against the Spike protein of SARS-Cov-2 (11) where GFP, rather than truncated EGFR, is used as reporter protein. The human T-lymphoblastic Jurkat cell line was transduced with a lentiviral vector that allows stable and coordinated co-expression of native S and GFP proteins from a monocistronic mRNA (**Figure 1A**). For each individual test, Jurkat-S-GFP cells were stained with a 1:50 dilution of serum sample followed by anti-human IgG1 PE, anti-human IgM Pacific blue and anti-human IgA AF647. Dead cells were excluded with the viability dye 7AAD (**Figure 1B**). Pools of positive and negative sera were used as controls to obtain two values for each Ig isotype, both based on the fluorescence of Ig anti-S and GFP: score and ratio. A Contour plot of the resulting staining with positive and negative control sera as well as the score and ratio values are shown in **Figure 1B** as a representative result. The score is calculated by applying an algorithm based on the proportional distribution of both fluorescences and stablishes a cut-off to discriminate positivity (0.095 for IgG1, 0.081 for IgA and 0.11 for IgM). The main contributor to this value comes from the slope of the linear adjustment of the fluorescence intensities of anti-S and GFP, which is positive for a seropositive sample (blue line in contour plots in **Figure 1B**). The Ig anti-S to GFP mean fluorescent intensity ratio is used as a relative quantitative value as it correlates with the titer and affinity of specific antibodies to S (11). This raw value is then normalized to 10 with the positive control serum (all Ig ratio data throughout this study are referred to the normalized value). The equivalence of the Ig ratio parameter into the international standard anti-SARS-CoV-2 immunogloblin BAU/ml (BAU, Binding Antibody Unit) titer was calculated using a calibrated standard (NIBSC 21/234, **Supplementary Figures 1A, B**). As a result, positive control serum was determined as 3548 anti-Spike IgG1 BAU/ml (note that this value is underestimated as we are detecting only IgG1 and not total IgG). Furthermore, serial dilutions of the positive control serum established the limit of detection of the JFCI into 0.333 anti-Spike IgG1 BAU/ml (**Supplementary Figure 1C**). International units for IgA titer could not be determined as a working standard for IgA was not available.

**Figure 2:**
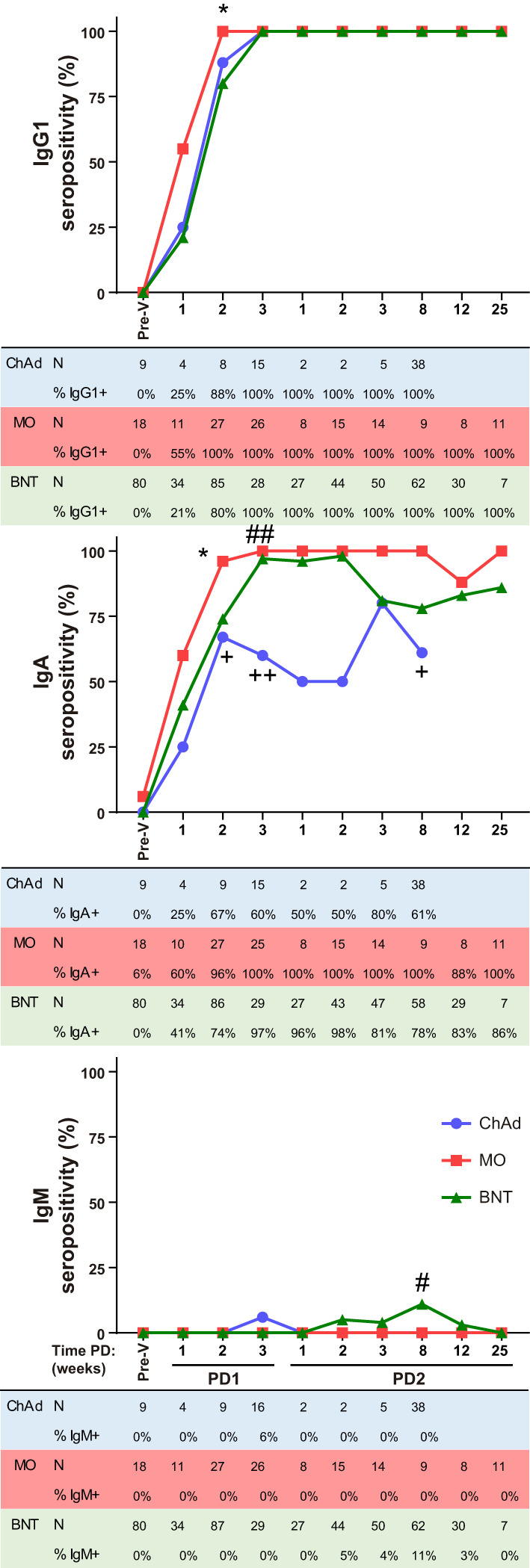
Ig anti-Spike seropositivity follow-up after vaccination. IgG1, IgA and IgM seropositivity in ChAdOx1 (ChAd), mRNA-1273 (MO) and BNT162b2 (BNT) vaccinated donors evaluated with the JFCI pre-vaccination (Pre-V) and at different time periods after the first (PD1) or the second (PD2) dose of the vaccines. Percentage of positive and total number of samples are depicted in tables below graphs. Samples were collected as 1 wk, 1-7 days; 2 wk, 8-16 days; 3 wk, 17-28 days; 8 wk, 29-60 days; 12 wk, 61-90 days; 25 wk, 91-174 days. Statistic comparisons were carried out for matched time PD between vaccines: +, ChAd vs MO; #, ChAd vs BNT; *, MO vs BNT. P values were determined using two-tailed Fisher’s exact test. The number of symbols indicates the p value summary: no symbol, no significance; ^+^, ^#^, *, p<0.05; ^++^, ^##^ p≤0.01.

**Figure 3:**
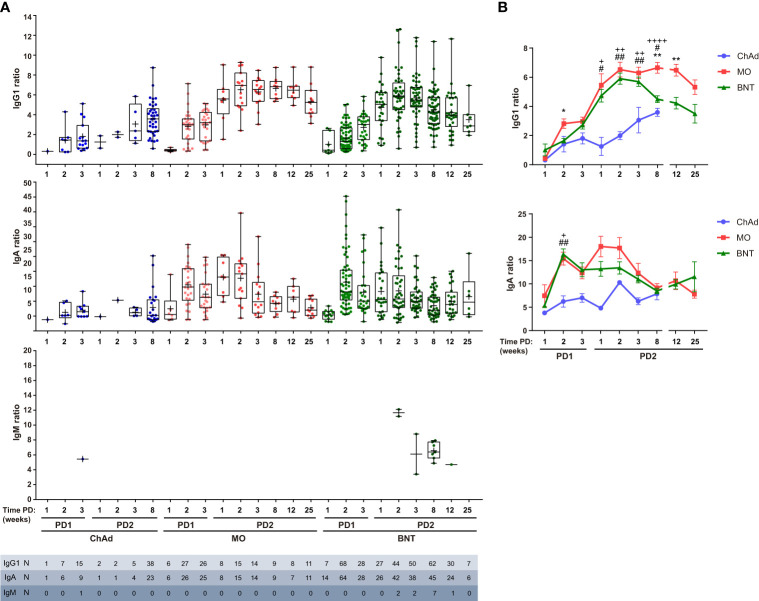
Ig anti-Spike titer follow-up after vaccination. **(A)** IgG1, IgA and IgM titers of seropositive samples described in **Figure 2**, expressed as Ig to GFP ratio. Median, line inside boxes; mean, +; bars, min and max values. **(B)** Overlay representation of data showed in **(A)** Mean and SEM indicated. Two-way ANOVA was used to compare data series starting 1 week PD1 and ending 8 weeks PD2 (IgG1, p < 0.0001 for both time PD and vaccine type variables) (IgA, p ≤ 0.01 for vaccine type variable). Multiple comparisons for matched time PD, are depicted in the graphs: +, ChAd vs MO; #, ChAd vs BNT; *, MO vs BNT. The number of symbols indicates the p value summary: no symbol, no significance; +, #, *, p<0.05; ++, ##, ** p≤0.01; ++++ p<0.0001. Two-way ANOVA with Sidak’s correction.

Using this method, we have performed a comparative analysis of humoral SARS-CoV-2-specific immune response in ChAdOx1 (Oxford-AstraZeneca, ChAd), mRNA-1273 (Moderna, MO) and BNT162b2 (Pfizer-BioNTech, BNT) vaccinated individuals. The experimental design of the study is depicted in **Supplementary Figure 2**, A first cohort was composed of a total of 682 individual samples within which 59 ChAd, 36 MO and 165 BNT vaccinated donors at different time-points (**Supplementary Table**). Reported SARS-CoV-2 positive PCR donors previous to vaccination were not included in this analysis. All donors became IgG1 seropositive after the vaccination was completed (post-dose 2, PD2) and seroconverted as soon as 3 weeks post-dose 1 (PD1) with the three vaccines (**Figure 2**). A significant, although reduced, proportion of seroconversion was detected after 2 weeks PD1 just with the BNT vaccine, with 80% IgG1 seropositivity. In terms of potency, ChAd vaccine generated the lowest IgG1 titer, followed by BNT, and being MO the highest, since significant differences in IgG1 ratios were detected among the three groups at the comparable time-point group of 8 weeks PD2 (**Figure 3**). Likewise, MO vaccine induced the highest IgA seroconversion with 96% seropositivity at 2 weeks PD1 compared to 67% and 74% for ChAd and BNT respectively (**Figure 2**). It should be noted that not all ChAd and BNT donors became IgA seropositive at PD2. ChAd induced the lowest IgA titers, whereas MO and BNT reached similar values (**Figure 3**). Regarding IgM, barely 11% of those vaccinated with BNT resulted positive at 8 weeks PD2 (**Figure 2**), being this isotype the less effectively detected, and reporting the lowest titers (**Figure 3**) in all the groups.

In terms of kinetics, IgG1 titer increased at PD2 compared to PD1, reaching its maximum level at 3 weeks after the second dose for MO and BNT vaccines and declined in a slower fashion compared to IgA that peaked at 2 weeks PD1 for MO and BNT, declined and reached a second peak at 1 week PD2 for MO and bit later for BNT (**Figure 3**). In spite the reduction observed, both isotypes remained quite stable at least up to the latest timepoint analyzed (25 weeks for MO and BNT). ChAd showed delayed kinetics reaching the maximum IgG1 and IgA titers at PD2.

**Figure 4:**
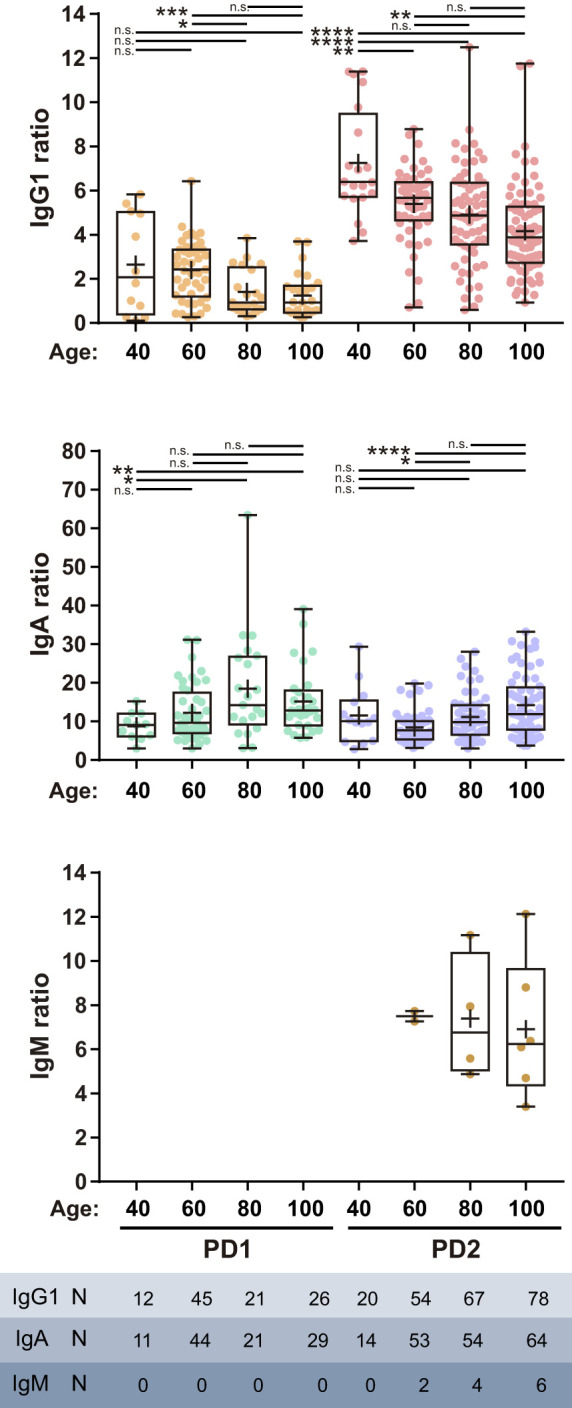
Effect of the age of the donor in the humoral response. IgG1, IgA and IgM titers of seropositive samples collected after the first (PD1) or the second (PD2) dose of BNT vaccine. Samples were grouped by the age of the donor as 40, ≤ 40; 60, 41-60; 80, 61-80; 100, 81-100 year-old. Median, line inside boxes; mean, +; bars, min and max values. Independent one-way ANOVA was used to compare PD1 and PD2 data series (IgG1 PD1, p = 0.0091; IgG1, PD2, p < 0.0001; IgA PD1, p = 0.0182; IgA PD2, p = 0.0002). IgG1 PD1, IgA PD1, IgA PD2 groups, Brown-Forsythe ANOVA and multiple comparisons with Games-Howell’s correction. IgG1 PD2, one-way ANOVA with Sidak’s correction. n.s., no significance; *p <0.05; **p ≤ 0.01; ***p ≤ 0.001; ****p < 0.0001.

There was a significant difference in the age of donors at 8-25 wk PD2 between MO and BNT groups (**Supplementary Figure 3**). The BNT donor group, derived mostly from nursing homes, were older on average than MO for the PD2 time-frame, and this difference in age could have an effect in the magnitude of the humoral response. For this reason, we next analyzed the effect of age at the time of vaccination on the humoral response. We studied this effect within the larger cohort of samples, BNT, in which there was also a broad representation of donor ages. Sample-data were pooled as PD1 or PD2 and grouped by age in 20 year-intervals. Similar proportion of individuals became IgG1 or IgA seropositive both at PD1 or PD2 independently of the age (**Table 1**). However, IgG1 titers were significantly reduced in older seropositive donors both after the first and after the second dose (**Figure 4**). By contrast, IgA titers increased with age. Similar results were obtained when data collection was restricted to 2-3 wk PD to avoid a possible bias due to differences in time PD (**Supplementary Figure 4**). Interestingly, IgM seropositivity was only found within the groups of older donors and after the second dose of vaccine (PD2) (**Table 1** and **Figure 4**). In those donors IgM was not produced in detriment of IgG1 (**Supplementary Figure 5A**). In fact there is a direct correlation between IgM and IgG1 but not between IgM and IgA titers for all the IgM seropositive samples (**Supplementary Figure 5B**). We also tested whether the donor’s gender had an influence in the humoral response but no difference was observed between male and female donors (**Supplementary Figure 6**).

**Table 1:** Total number and proportion of positive samples for IgG1, IgA and IgM after the administration of one or two doses grouped by the age of the donor.

		IgG1		IgA		IgM			
		n	% POS	n	% POS	n	% POS		
BNT	40 PD1	15	80	14	79	15	0		
	60 PD1	69	65	68	65	69	0		
	80 PD1	26	81	27	78	27	0		
	100 PD1	38	68	39	74	39	0		
	40 PD2	20	100	20	70	20	0		
	60 PD2	54	100	54	98	55	4		
	80 PD2	67	100	64	84	67	6		
	100 PD2	78	100	77	83	78	8		
Comparison (Fisher’s exact test, two-tailed)								P-value	P value summary
IgA POS PD2						40 vs 60		0,001	**
IgA POS PD2						60 vs 80		0,011	*
IgA POS PD2						60 vs 100		0,008	**

Non-statistical differences were found between other age-groups for any vaccine.

POS, positive; 40, 60, 80 and 100 aged groups described in **Figure 4**.

*p < 0.05; **p < 0.01.

**Figure 5:**
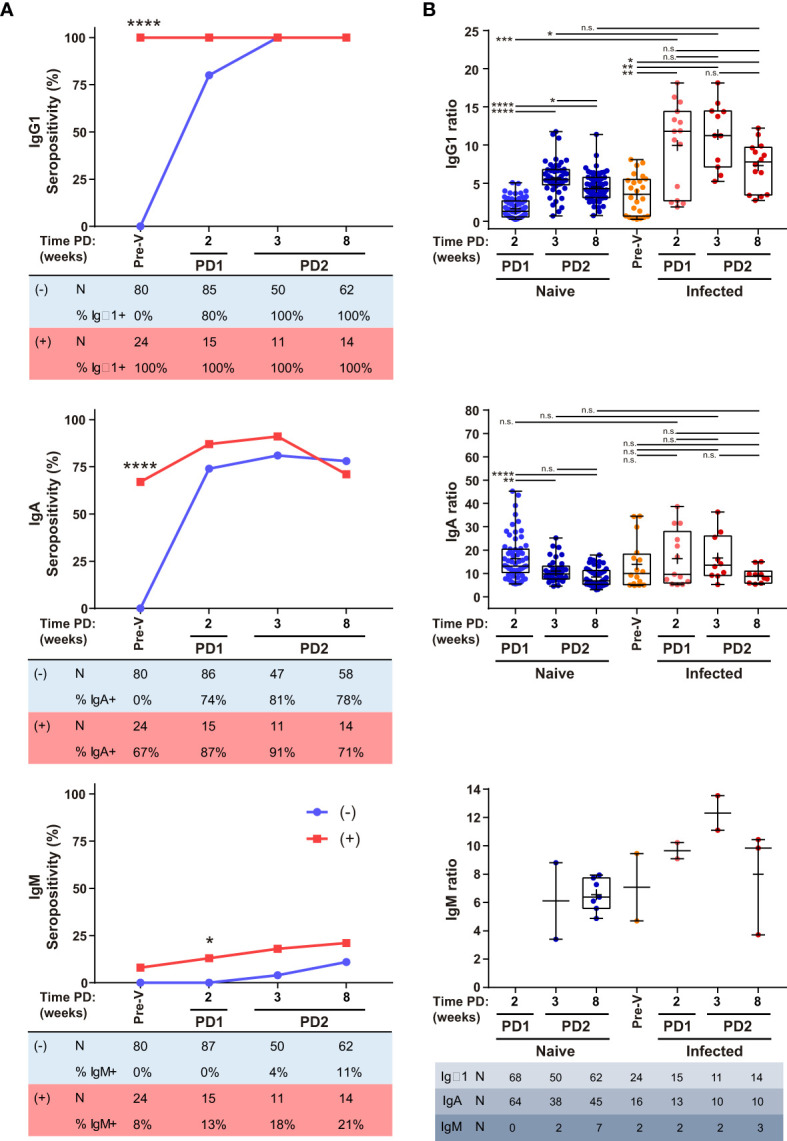
Humoral response in previously infected donors. IgG1, IgA and IgM seropositivity **(A)** and titers **(B)** of naïve (-) and previously infected and IgG1 seropositive (+) donors vaccinated with BNT. Total number and percentage of positive samples are shown in tables below seropositivity graphs. Sample collection time as described in **Figure 2**. Median, line inside boxes; mean, +; bars, min and max values. P values were determined using two-tailed Fisher’s exact test **(A)** and Brown-Forsythe ANOVA with Games-Howell’s correction **(B)**. n.s., no significance; *p < 0.05; **p ≤ 0.01; ***p ≤ 0.001; ****p < 0.0001.

A second cohort of 64 samples from 25 donors that had been previously infected with SARS-CoV-2 (and were IgG1 seropositive) prior to vaccination were evaluated after one or two doses of BNT compared to the first cohort of naive donors (**Supplementary Figure 2** and **Supplementary Table**). We found that one dose of BNT administered to previously infected individuals was sufficient to induce the maximum level of IgG1 response (**Figure 5B**). Furthermore, the humoral response did not increase after the second BNT dose in this group, unlike the group of naive individuals who had not been infected before vaccination. Nearly two-thirds of infected donors were already IgA seropositive prior to vaccination (**Figure 5A**). The magnitude of the IgA response reached similar levels in both groups, although it was transiently sustained in previously infected donors decreasing to similar levels at 8 wk PD2 (**Figure 5B**). Strikingly, IgM seropositive donors, although scarcely detected as occurred for the first cohort, were also detected in previously infected donor not only at PD2 but also at PD1 and pre-vaccination (**Figures 5A, B**).

## Discussion

Here we have performed a longitudinal study to compare the humoral response to three different vaccines against SARS-CoV-2 in a cohort of Spanish individuals. We show that although the three vaccines eventually induced an efficient humoral response against the Spike protein of the virus, some differences in antibody seroconversion and titer were found. Seroconversion to IgG1 was fast with the three vaccines, reaching 100% seropositivity even only after one dose. All donors vaccinated with mRNA-1273 or BNT162b2 seroconverted to IgA whereas not all donors receiving the ChAdOx1 vaccine did. In the case of IgM only a minor seroconversion was detected in elderly donors vaccinated with BNT162b2. In terms of antibody titer, ChAdOx1 vaccine induced lower IgG1 and IgA titers whereas similar values were obtained for mRNA-1273 and BNT162b2. The differences in titers observed between the two mRNA vaccines could be age-related (discussed below), as the mean age of donors for the BNT group is higher (**Supplementary Figure 3**). In summary, our data indicate that mRNA-based vaccines, mRNA-1273 and BNT162b2, provoke a more potent humoral response and could be more immunogenic than the adeno-based ChAdOx1. Up to now only few studies have compared the humoral response of vaccinees in parallel using the same method (14–16): (i) Fabricius et al. found no difference in IgG and IgA titers PD1 between MO and BNT and increased IgA production at PD2 for MO. IgG and IgA titers were higher compared to ChAd but the latter received only one dose. Interestingly ChAd/MO and ChAd/BNT heterologus regimes for full vaccination induced higher IgG and IgA titers than ChAd/ChAd full vaccination. (ii) Lechosa-Muñiz et al. analyzed only one timepoint after full vaccination with mRNA vaccines or a single dose of ChAd and although they did not found a statistical difference between MO and BNT IgG titers, again they were higher compared to ChAd. (iii) Neumann et al. analyzed only total IgG and found also a trend in higher titers for MO compared to BNT, PD1 or PD2. They also analyzed one dose for ChAd, giving the lowest titers. Our results are in accordance with these studies but expand the analysis to a complete vaccination regime.

The high sensitivity of the JFCI detection method allows not only the detection of low IgG1 titer but also the detection of IgA in serum. Most IgA antibodies are present in mucosal tissues. Total IgA is usually around 8 times (in average, it can be up to 40 times) less abundant in serum than IgG (total serum IgG range 7.5-22 mg/ml; total IgA range 0.5-3.4 mg/ml) and moreover usually only specific IgG and IgM are detected by ELISA/CLIA. It will be interesting to study if specific serum IgA detection could correlate with a proper IgA response in body cavities that would be essential to impede infection, prevent transmission or protect from disease severity. In fact IgA antibody responses have been detected in nasal fluids of patients infected with other coronaviruses, and were associated with shortened periods of viral shedding (17). Elevated levels of IgA has also been associated with influenza vaccine efficacy (18, 19). In this way serum IgA testing could serve as a correlate for protection.

We were not able to efficiently detect high levels of serum IgM. This could be due to low affinity of IgM antibodies. IgM is the first isotype rapidly secreted during the humoral response, mostly of low-affinity, followed by the production of high-affinity antibodies of different mature isotypes. The JFCI method is based on low protein expression on the Jurkat cell surface, allowing the detection of high-affinity antibodies like IgG1 or IgA isotypes. On the contrary, ELISA is based on high concentration of recombinant protein fragments allowing the detection not only of high but also of low affinity antibodies. Higher epitope density likely allows for monogamous multivalent binding of low affinity antibodies. Other immunoassays to detect specific anti-Spike antibodies by flow cytometry have been developed and either do not show IgM detection (20, 21) or they do efficiently detect IgM on beads where the antigen has been adsorbed (22–24) or on the surface of HEK-293T cells where the antigen is overexpressed (25–28). In these two approaches epitope density is higher compared to the Jurkat-Spike cells of the JFCI, in line with our argument.

In terms of IgG1 and IgA kinetics our results show that humoral response to vaccination parallels the response to natural infection. It has been reported that there are concomitant waves of IgA, IgM and IgG production in COVID-19 patients, being IgA and IgM cleared faster than IgG (29, 30). We have found an increase in IgA after each of the immunizations, reaching a peak around 2 weeks after priming or boosting immunizations and decreasing afterwards. On the contrary, IgG1 increased progressively reaching its maximum level around 2-4 weeks after the second, boosting, dose and decreasing slightly afterwards. Of note, ChAd vaccine induced delayed kinetics, highlighting a decreased potency compared to mRNA vaccines. For both isotypes, seropositivity is stabilized and maintained at least during the time period of this study, more than 6 months after the boosting immunization. Studies after this time-frame are ongoing in order to know how long the response to vaccines persist. For IgM, as the JFCI does not allow detection of low affinity antibodies, we cannot compare responses to vaccines and natural infection. Nonetheless it is interesting to find that some individuals produced high-affinity IgM a long time after immunization. Of note, humoral response in vaccinated individuals reached at least the same titer as natural infection.

Immunogenicity in the elderly has also been a matter of concern. The immune system efficacy is affected by age, so a reduced humoral response could be expected in older vaccinated individuals. Seroconversion rate was not affected by age although we found an inverse correlation between age and the magnitude of the IgG1 response. It would be also interesting to correlate the magnitude of the humoral response and protection since none of the individuals analyzed remained IgG1 seronegative after the complete vaccination. It is also noteworthy that IgA titers, unlike IgG1 ones, increased with age and also that a few IgM seropositive cases were detected only after PD2 in older individuals as well as in the infected pre-vaccination group (in both cases the mean age of IgM seropositive individuals were 80 year-old). We did not find any relationship between IgM and IgA response (**Supplementary Figure 5B**) and all IgM+ donors were also IgG1+ (**Supplementary Figure 5A**). These observations reveal some kind of abnormality caused by age. A possible explanation could be a bias in isotype switching caused by alterations in the germinal center or an imbalance in long-lived plasma cells secreting IgA homing to mucosae. It could be also possible that memory against certain coronaviruses occurring only in the elderly could induce the generation of high affinity IgM. A decline in IgG antibody titers with age has also been observed after the second dose of mRNA vaccines (31, 32). Although the cohort analyzed in Goel et al. was not enriched in subjects over 50, they also found a clear reduction in memory B cells with age. Other studies have reported a negative association between vaccine-induced antibody titers and age after a single dose of mRNA vaccines (33–36). A limitation of our study is that it was conceived as a retrospective study, what makes it non-randomized, so other unknown variables as co-morbidities or donor’s medication were not included in the analysis and could not be excluded as factors influencing the immune response.

Finally, we show that previously seropositive individuals required only one dose of the vaccine to reach the maximum humoral response, reaching indeed higher IgG1 titers than full-vaccinated donors. This result is in line with previous studies (31, 37–39), and brings to light the importance of saving doses that would be unnecessarily administered to previously infected people. It would be more reasonable to reserve boosting to new versions of vaccines adapted to emerging variants of concern. But these decisions on vaccination should be taken with care because some people previously infected could mount a weak immunological response, especially those that do not develop COVID-19 symptoms, likely leaving these individuals with suboptimal protection. Diagnostic antibody testing would be required to take the more convenient decision, for which our JFCI would be of great usefulness. Moreover, serum IgG1 is the more informative isotype to test since it is the most persistent isotype present in blood.

Studies of the type herein described, analyzing longer times post-vaccination, and extended to other vaccines and vaccine-combinations, will be very useful to know the duration of the humoral response against SARS-CoV-2 in order to design the protocol for new boosting immunizations to sustain the response without wasting valuable doses. This will help to determine the better efficacy-cost ratio for a more suitable distribution of vaccine doses. In addition, prospective studies on vaccinated population testing not only humoral but also cellular responses will be required as a correlate for protection after infection with current and emerging viral variants. This will help to determine the degree of humoral response required for protection from infection and/or development of different degree of COVID-19 symptoms, a tool especially important to prove the efficacy of different vaccines and for the design of new vaccines.

## Data Availability Statement

The original contributions presented in the study are included in the article/**Supplementary Material**. Further inquiries can be directed to the corresponding author.

## Ethics Statement

Serum sample collection was included in the study “ACE2 as a biomarker with utility for identification of high risk population for SARSCoV-2 infection and prognosis of evolution in COVID-19” approved by Autonomous University of Madrid Research Ethics Committee, no.2352. The patients/participants provided their written informed consent to participate in this study.

## Author Contributions

SR-P, MQ, LH, and DA performed research and analyzed the data. AO and SA-F provided clinical samples, the original idea and edited the manuscript. BA analyzed data and supervised research. PD supervised and designed research, analyzed data and wrote the manuscript. All authors contributed to the article and approved the submitted version.

## Funding

This work was funded by intramural grant CSIC-COVID19-004: 202020E081 (to BA). L.H has been supported by an FPI fellowship from the Spanish Ministry of Science and Innovation.

## Conflict of Interest

The authors have issued a patent application owned by CSIC. Authors SR-P, MQ, SÁ-F, and AO are employed by VITRO S.A.

The remaining authors declare that the research was conducted in the absence of any commercial or financial relationships that could be construed as a potential conflict of interest.

## Publisher’s Note

All claims expressed in this article are solely those of the authors and do not necessarily represent those of their affiliated organizations, or those of the publisher, the editors and the reviewers. Any product that may be evaluated in this article, or claim that may be made by its manufacturer, is not guaranteed or endorsed by the publisher.
